# A national Programme Budgeting and Marginal Analysis (PBMA) of health improvement spending across Wales: disinvestment and reinvestment across the life course

**DOI:** 10.1186/1471-2458-14-837

**Published:** 2014-08-12

**Authors:** Rhiannon Tudor Edwards, Joanna M Charles, Sara Thomas, Julie Bishop, David Cohen, Sam Groves, Ciaran Humphreys, Helen Howson, Peter Bradley, on behalf of the Public Health Wales Health Improvement PBMA team

**Affiliations:** Centre for Health Economics & Medicines Evaluation, Ardudwy, Normal Site, Bangor University, Bangor, Gwynedd, UK; Public Health Wales, Hadyn Ellis Building, Maindy Road, Cardiff, UK; Health Economics and Policy Research Unit, University of South Wales, Pontypridd, UK; Public Health Wales Observatory, Building 1, St. David’s Park, Job’s Well Road, Carmarthen, UK; Public Health Wales, 14 Cathedral Road, Cardiff, UK

**Keywords:** Programme budgeting, Marginal analysis, Disinvestment, Investment, Public health, Health economics

## Abstract

**Background:**

Wales faces serious public health challenges, with relatively low life expectancies and wide inequalities in life expectancy with associated pressures on the National Health Service (NHS) at a time of financial recession. This has led to growing recognition of the need to better understand the range of health improvement and prevention programmes across Welsh Government, NHS, local government and voluntary sector agencies.

**Methods:**

The Minister for Health and Social Care commissioned Public Health Wales, the single national public health organisation, to establish a Health Improvement Advisory Group, to oversee a Programme Budgeting and Marginal Analysis (PBMA) expert panel. The panel drew on evidence from a range of sources to explore potential alternative modes of health improvement initiative delivery across Wales. Electronic voting was used to agree an appropriate time horizon for health improvement programme outcomes, main objective of the health improvement review and criteria for evaluating candidate services for disinvestment and investment. The panel also used electronic voting to state whether they wished to disinvest or invest in a candidate service.

**Results:**

The review identified a budget of £15.1 million, spanning 10 Welsh Government priority areas, and 6 life course stages. Due to lack of evidence the panel recommended total disinvestment in 7 out of 25 initiatives releasing £1.5 million of resources, and partial disinvestment in a further 3 interventions releasing £7.3 million of resources. The panel did not recommend increasing investment in any of the 25 initiatives under review. Marginal analyses prioritised child health, mental health and wellbeing and tobacco control as key areas for investment.

**Conclusions:**

Wales is championing a concept of “prudent healthcare”. The PBMA exercise undertaken here was a transparent evidence-based tool to reach decisions about potential for disinvestment and reinvestment in health improvement strategies. It also demonstrates the potential wider application at a national level across government public health functions, to ensure resources are most cost-effectively deployed, with due consideration for equity.

**Electronic supplementary material:**

The online version of this article (doi:10.1186/1471-2458-14-837) contains supplementary material, which is available to authorized users.

## Background

### Public health challenges in Wales

Despite an increase in healthy life expectancy in Wales in recent years, local authorities continue to experience among the worst life expectancies in the UK, and the gap between the most and least deprived remains wide. Smoking causes about 1 in 5 deaths in Wales [1]. Prevalence is currently 23% and is highest in young Males aged 25–34 at 38% [1]. About 45% of the population drink above guideline amounts of alcohol, over 1000 people a year die from alcohol in Wales, and there are over 55,000 hospital admissions due to alcohol in Wales per year [2]. Only about a third of adults eat 5 fruit and vegetables a day, under a third meet physical activity guidelines with about 30% of the adult population taking no exercise in a typical week [2]. As a consequence of unhealthy eating and low physical activity, 57% of the Welsh adult population is overweight or obese [2]. There is a growing need from policy makers for interventions that address the above challenges, but are also considered a good use of public resources. The cost-effectiveness evidence base for public health interventions is beginning to grow.

### Growing evidence of the cost-effectiveness of public health interventions

Owen et al., 2011 have synthesised the evidence of the cost-effectiveness of public health interventions underpinning National Institute for Health and Care Excellence (NICE) Public Health Guidance from 2006–2010 [3]. They analysed 200 base-case cost-effectiveness estimates. Findings showed the majority of public health interventions assessed were highly cost-effective, 85% of which had an incremental cost-effectiveness ratio less than £20,000 per Quality Adjusted Life Year (QALY) and 89% at the higher threshold of £30,000 per QALY [3]. The authors conclude that the next step would be to develop a framework that allows the combination of economic analysis and other criteria to support local decision makers to make better investments. Although there is a need for quality evidence from Randomised Controlled Trials (RCTs) that pay particular attention to the challenges of conducting economic evaluations of complex (as defined by the Medical Research Council (MRC)) [4] public health interventions as recommended by Kelly et al. (2005), McDaid & Needle, (2008) and Weatherly et al. (2009), [5–7], there is also a need for expert opinion and common sense. Programme Budgeting Marginal Analysis can be employed as a means of using expert opinion as a part of evidence based decision making.

### Programme Budgeting and Marginal Analysis (PBMA)

Programme Budgeting and Marginal Analysis (PBMA) is a process that helps decision-makers maximise the impact of healthcare resources on the health needs of a local population or meet other specified goals such as equity considerations. Programme budgeting is an appraisal of past resource allocation in specified programmes, with a view to tracking future resource allocation in those same programmes. Marginal analysis is the appraisal of the added benefits and added costs of a proposed investment or the lost benefits and lower costs of a proposed disinvestment [8, 9]. Some programmes can absorb a degree of contraction, whilst still continuing e.g. through better targeting. It is important to be aware of the links across programmes and, therefore, how changes in expenditure on one programme may impact on others. The PBMA process requires information on expenditure by programme for example, by an annual budget and/or numbers of full time equivalent posts (WTE). The stages of a PBMA exercise are shown in Table 1 below.

**Table 1:** **The eight stages of PBMA by Brambleby and Fordham**

Stage	Description
1	Choose a set of meaningful programmes/initiatives.
2	Identify current activity and expenditure in those programmes/initiatives.
3	Think of improvements.
4	Weigh up incremental costs and incremental benefits and prioritise a list.
5	Consult widely.
6	Decide on changes.
7	Effect the changes.
8	Evaluate progress.

A recent review considered factors that may explain the success or otherwise of PBMA exercises [10]. Tsourapas & Frew (2011) found 28 applications of PBMA spread across the UK, Australia, New Zealand and Canada [10]. Findings showed PBMA was successful in 52% of cases where success was defined in terms of the participants gaining a better understanding of the area under interest [10]. PBMA was successful in 65% of cases where success was defined as ‘implementation of all or some of the PBMA panel’s recommendations’ [10]. Forty-eight percent of the studies were successful where success was defined in terms of disinvesting or resource reallocation; and in 22% where success was defined in terms of adopting the framework for future use [10]. The authors concluded that the definition of success influenced the rate of successful PBMA applications. They argue for a broadly accepted definition of success to allow greater comparability within the field [10].

There has also been more recent use of PBMA as a framework for disinvestment [11]. When conducting a rapid review of applied PBMA framework, we found papers describing PBMA exercises of maternity services [12], Surgical Department [13], gynaecology services [14] and GP led community hospital care for stroke patients [15]. This paper describes a national PBMA exercise of the annual health improvement budget of the Welsh Minister for Health and Social Care. We believe this to be the first published description of a PBMA exercise at a national level [16].

## Methods

We describe below the process of conducting the PBMA exercise, with particular reference to the perspective, development of the panel, gathering of evidence and the marginal analysis task.

### Perspective of the health improvement review

This PBMA exercise was completed on behalf Public Health Wales and considered public health interventions at a national level, taking into account NHS services and those provided by public and private partners. This PBMA exercise assessed the health improvement budget of the Minister for Health and Social Care. At the beginning of the process health improvement was defined under the Ottawa Charter (1986) [17]. This definition of health improvement highlights the importance of reorienting health services, creating supportive environments, improving personal skills, community action, and the role of healthy public policy. Once the perspective was established, a panel was established to review the evidence gathered and reach the disinvestment/investment decisions.

### Development of a PBMA panel

An expert panel list of 30 potential members was established with representatives from: Public Health Wales, Welsh Government, NHS Health Boards, third sector, local government and primary care. Each member of the suggested panel was sent an invitation to participate in the PBMA exercise by e-mail. The e-mail described the purpose of the exercise, the invited member’s role in the panel, the commitment required and the dates and times of the proposed meetings. The invited members were able to decline to participate, and panel members who agreed to participate were able to withdraw their membership at any time. As this was a government initiated evidence based, decision making exercise ethical approval was not required.

Once the budget was defined, the researchers were informed that the budget contained 25 initiatives, accounting for the total £15.1 million of the Minister’s spend in health improvement across Wales. The panel drew upon evidence collated for each initiative from the review sub-groups, stake holder consultation and an NHS/ primary care sub-group to explore potential alternative modes of health improvement delivery across Wales. The PBMA panel met three times and the sessions were facilitated by a session leader. The sessions started with an explanation of the exercise and the review of evidence undertaken. A discussion of the criteria to appraise the evidence was also conducted with a list of six final criteria agreed by the panel. These criteria were as follows: considered a priority health issue for Welsh Government, opinions of experts, stakeholder views, presence and robustness of evidence of effectiveness, presence and robustness of evidence of cost-effectiveness and impact or potential impact on reducing inequalities in health. The options above were used as part of the electronic voting exercise. The panel were then asked to vote electronically on the preferred objective of the Health Improvement Review, the top four criteria for the health improvement review from 12 PBMA panel members and to agree the most relevant time horizon for this PBMA exercise. The vote was conducted and then the results were displayed electronically using graphics and discussed, with an opportunity to revote if required. The panel were given the evidence underpinning each of the initiatives to read between the second and third session. At the third session, the panel were asked to vote for candidates for investment and disinvestment. The vote was conducted; the results were displayed and discussed, with an opportunity to revote if required.

### Boundaries of the programme budget

This programme was a historically determined programme budget of Ministerial resources currently devoted specifically to health improvement at an All Wales level. There are other resources known to be used for health improvement purposes, sometimes matched with Local Government or voluntary sector spending. However, these were considered outside the remit of this analysis.

The review established five operational sub-groups to support the review, as outlined below. Information from each of these groups was summarised and then combined and collated into summary booklets for each initiative. During this summary process the review teams decided that a scoring system was required to help guide the panel. A traffic light system was agreed as the most appropriate and visually effective structure. Each of the evidence review sub-teams applied a traffic light rating system to their particular stream of evidence and then an overall traffic light grading was assigned to each initiative based upon all the available evidence gathered by the sub-groups. The overall traffic light grading was as follows: Red – based on published evidence and consultation, this intervention is unlikely to bring a population health benefit and alternatives should be explored to achieve these health goals. Amber - greater evidence needs to be found for the impact of this initiative at a population level and/or there are elements of the programme that need substantial revision or there is insufficient evidence available to make a judgment. Green - this is a sound programme with a reasonable evidence base; however, we need to ensure that reach is maximised and it is cost effective. See Additional file 1 for a description of the evidence booklet methodology. These booklets were distributed to the PBMA panel members for their consideration before the sessions.

### Protocol for review of effectiveness evidence

A pragmatic search strategy was designed using specified health databases (NHS Evidence, Cochrane Collaboration, Cambell Collaboration, Health Evidence Canada) and the search-engines (PubMEd and Google Scholar). For initiatives where recent high quality secondary analyses of the primary literature were found, searches were narrower and terminated at an earlier stage. Searches for questions that yielded little high quality data initially were broadened by date or by search terms in an attempt to capture related work. Retrieved articles were screened for inclusion by two independent reviewers (disagreements resolved by discussion), on the basis of direct relevance to the initiative or component interventions and type of article, thus single studies were not included if higher level evidence was available. Evaluations of interventions in practice were also sought and interventions were assigned an overall evidence rating taking into account potential and actual evidence of effectiveness.

### Protocol for review of cost-effectiveness evidence

Relevant articles identified from an evidence search (2002–2012) of NICE, Pub-Med and the Centre for Reviews and Dissemination (CRD) Database using key terms from each of the 25 initiatives were sourced and then appraised. Evidence was defined as; *directly* relevant i.e. an economic evaluation of a specific intervention delivered through the programme/initiative stated in the list of included programmes; or *indirectly* relevant (where directly relevant evidence is unavailable) i.e. evaluation of related intervention similar to the one delivered through the programme/initiative or as part of the intended aims of the programme/initiative stated in the list of included programmes by either method of delivery (school-based smoking cessation) or target population (pregnant women). The Drummond et al. (2005) checklist for a sound economic evaluation was used to appraise evidence found in the electronic searches [18]. A subjective judgement of the overall balance of economic evidence was made by the economic evidence sub-group and a traffic light system of grading was used.

### Stakeholder consultation process

The review involved wide consultation to gain opinions of stakeholders such as practitioners delivering the initiatives and the public who may have come into contact with particular initiatives. These were used in the evidence booklets to give the panel an indication of stakeholder views. This was undertaken as part of a wider “change management” strategy to ensure the public had opportunity to discuss any possible changes to services and relay their concerns so policy makers could understand the potential impacts. The consultation process involved a range of engagement events including; visits to local public health teams across the 7 health boards, these teams were often involved with the delivery of health improvement programmes, visits with Public Health Wales staff. Beaufort Research were commissioned to undertake a public survey and to conduct six focus groups and six in-depth family interviews. Eight consultation events were held across Wales addressing the initiatives with regards to different stages of the life course. An open online feedback form was hosted on the bilingual review web pages on the Public Health Wales website, to further engage the public and staff. Responses were assigned a traffic light system based upon the overall majority of positive, negative and mixed feedback from each of the groups.

### Equity review

The extent to which each of the initiatives addressed equity concerns was also supplied in the evidence booklets. A traffic light categorisation system was developed to grade the degree of equality/equity focus of each initiative under review. Some of these initiatives have a degree of complexity which required explanation in addition to the traffic light grading. These include some where there has been a change of focus since inception and others where programme employees act as intermediaries and local areas are largely autonomous in the way initiatives are delivered. It should be noted that the categories apply to the intention of the programme rather than the supporting evidence, effectiveness or cost-effectiveness, which have been reviewed separately.

### NHS/primary care consideration

The evidence booklets also detailed options for alternative modes of delivery through existing mainstream services. The mechanism of delivery was summarised with consideration given to alternatives where appropriate.

Those directly involved in the intervention delivery or commissioning of services were invited to correct matters of accuracy and supply additional evidence for consideration. This was reviewed and a final assessment agreed by the panel.

We went on to undertake a pragmatic high-level marginal analysis task as part of the PBMA process.

### Marginal analysis

Three months following the PBMA sessions all members of the PBMA panel and HIAG received a high level pragmatic marginal analysis electronic task and supporting document. The supporting document provided the recipients with a refresher of the PBMA sessions, including grading of evidence and the outcomes of electronic voting for criteria and investment/disinvestment decisions. As the panel did not wish to continue investing in any of the current programmes, based on available evidence, a high level task was developed in which the recipients were asked to consider what Public Health Wales should do with a hypothetical £5 million. This sum of money was chosen based upon the median amount of monies released from the recommended disinvestment and partial disinvestment decisions made by the panel. The panel and HIAG members were asked to rank, in order of importance, their top 3 priority areas (out of a choice of 11) and their top 3 life course stages (out of a choice of 6), in which new approaches to health improvement should be developed with this hypothetical £5 million. They were also asked to state how much of the £5 million they would allocate to each of their top 3 choices and to give a brief rationale for their choices.

## Results

### The programme budget

We identified 25 specific health improvement initiatives within the programme budget, see Table 2 below. See Additional file 2 for a brief description of each of the initiatives. There were a number of initiatives where no or little evidence was available. Economic evidence was sparse; with 11 of the 25 initiatives having no available evidence of cost-effectiveness, cost-utility or cost-benefit. A total of 12 panel members attended each of the 3 PBMA sessions.

**Table 2:** **The 25 initiatives identified in the PBMA exercise**

Initiative	Approx. spend 2012/13	Assessment category
**Cooking Bus**	£655 k	Red – Based on published evidence and consultation, this intervention is unlikely to bring a population health benefit and alternatives should be explored to achieve these health goals.
**MEND**	£480 k	
**Mental Health First Aid**	£143 k	
**Smokebugs**	£131 k	
**National Breastfeeding Programme – Breastfeeding Peer Support Programme (BPSP)**	£31 k	
**National Breastfeeding Programme – Breast-feeding Welcome Scheme (BFWS)**	£11 k	
**Health Challenge Wales website Cost**	£38 k	
**Smokers Helpline**	£30 k	
**Smoking Resources**	£30 k	
**Skin Cancer Awareness**	£15 k	
**Designed to Smile**	£3.75 M	Amber - Greater evidence needs to be found for the impact of this initiative at a population level. and/or There are elements of the programme that need substantial revision or There is insufficient evidence available to make a judgment
**Welsh Network of Healthy Schools Schemes**	£2.3 M	
**Stop Smoking Wales – Pre Surgery**	£2.2 M (NB total spend on SSW over 5 programmes as it was not possible to break spend down to individual programmes)	
**Stop Smoking Wales – Pregnancy**		
**Stop Smoking Wales – Vulnerable Groups**		
**Stop Smoking Wales – Brief Intervention Training**		
**Fresh Start Wales**	£700 k	
**Alcohol Brief Interventions in Primary Care Training**	£100 k	
**HIV Prevention**	£56 k	
**National Exercise Referral Scheme**	£3.5 M	Green - This is a sound programme with a reasonable evidence base however we need to ensure that reach is maximised and it is cost effective.
**Stop Smoking Wales – Adults**	£2.2 M (NB spend over 5 programmes as it was not possible to break spend down to individual programmes)	
**ASSIST**	£300 k	
**National Breastfeeding Programme - Baby Friendly initiative (BFI)**	£110 k	
**No Smoking Day**	£27 k	
**Teenage Pregnancy Pilot**	£150 k	White – a Pilot
**Steroids and Image Enhancing Drugs**	£50 k	White – insufficient information to make an assessment.
**Champions for Health**	£30 k	White – It is not clear what theoretical or evidence base has been used in planning this intervention. Without an evaluation (which specifies and measures primary outcomes) wider implementation cannot be recommended.

The 25 initiatives identified in the PBMA exercise with an overall traffic light grading and summary statement from the five evidence sub-group categories (total of £15 million expenditure).

Nineteen of the 25 initiatives included in the PBMA exercise received an overall evidence traffic light grading of red or amber, stating alternatives should be explored to achieve the health improvement goals outlined by the initiative or required further evidence. Fifteen of the 25 initiatives had no economic evidence.

### Spending by life course stage

**Figure 1:**
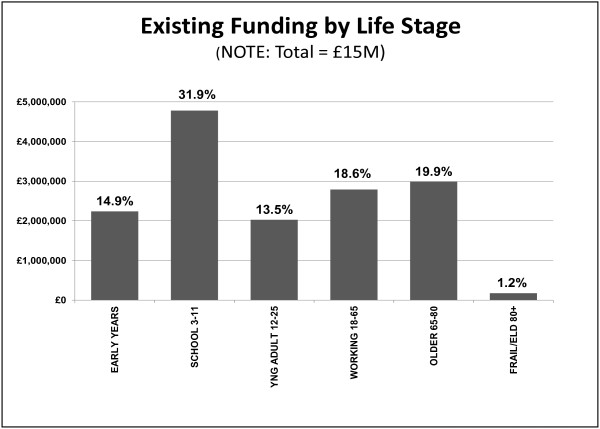
**Spending by life course stage of the 25 health improvement initiatives.** Spending on the 25 health improvement initiatives by each life course stage.

Figure 1 illustrates the prevailing spending per life course stage of the £15 million of the identified programme budget.

### Establishing criteria for evaluating the programme and candidate interventions for investment and disinvestment

The 12 PBMA panel members were asked, using electronic voting to identify criteria with which to judge the relative merit of candidate interventions for investment and disinvestment see Tables 3, 4 and 5.

**Table 3:** **Preferred objective of the health improvement review electronic vote results**

Objective	Percentage vote	n
A housekeeping exercise of current patterns of spending	8%	1
A means of bringing a culture of evidence based decision making into routine policy	42%	5
An academic exercise to explore the degree of success achieved in applying PBMA	8%	1
A means of bringing evidence of cost-effectiveness into resource planning	42%	5

**Table 4:** **The top four criteria for the health improvement review electronic vote results**

Criteria	Percentage vote	n
Stakeholder views	20%	2
Presence and robustness of evidence of effectiveness	34%	4
Presence and robustness of evidence of cost-effectiveness	27%	3
Impact or potential impact on reducing inequalities in health	19%	2

Results of the electronic vote for the top four criteria for the health improvement review from 12 PBMA panel members.

**Table 5:** **The most relevant time horizon to assess outcomes of the health improvement programmes under review**

Time horizon	Percentage vote	n
1 year	8%	1
5 years	50%	6
10 years	17%	2
15 years	8%	1
20 years	17%	2
Other	0%	0

Results of the electronic vote for the most relevant time horizon that should be used in this PBMA based review of health improvement programmes in Wales – it was stated to the panel in the session that this time horizon related to outcomes rather than the process of the review.

### Generating candidate initiatives for investment and disinvestment

**Figure 2:**
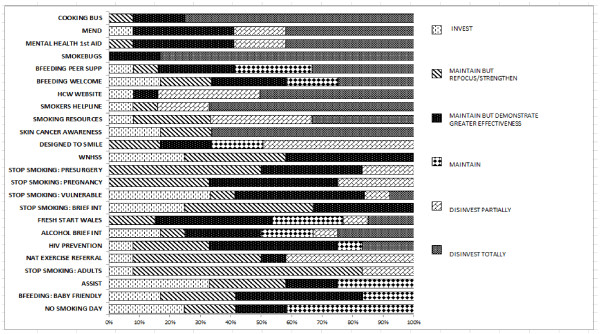
**Investment and disinvestment decisions made by the panel for each of the 25 initiatives.** Candidates for investment and disinvestment recommendations from votes made by the PBMA panel (n = 12) for the 25 initiatives under review. Please note the initiatives considered as pilots (Teenage Pregnancy Pilot, Steroids and Image Enhancing Drugs and Champions for Health) were not included in the voting.

Figure 2 illustrates that the PBMA panel was able to reach a majority vote to recommend disinvestment in 7 out of 25 initiatives releasing £1.5 million per annum (The Cooking Bus, Smoke Bugs, Skin Cancer Awareness, Health Challenge Wales Website, Mind, Exercise, Nutrition… Do it! (MEND), Mental Health First Aid and Smokers Helpline). Although the overall health improvement goals were rational, it was stressed that this was on the basis of a lack of evidence of effectiveness, cost-effectiveness or support from local public health teams, or any evidence of impact on inequality.

These results did not mean that the target stages of the life course e.g. primary school children, or the goal of limiting health harming behaviours were less important than other goals, rather that such goals should be addressed in other, evidence based ways e.g., environmental change. The PBMA panel also recommended partial disinvestment in a further three interventions releasing £7.3 million of resources, including some big spend areas such as Designed to Smile and National Exercise Referral Scheme. Because of a lack of published evidence at the time of effectiveness, cost-effectiveness and impact on inequalities, the panel did not vote in any majority fashion to invest further in any of the 25 interventions under review. Following this, a high level marginal analysis task was developed to assess which priority areas and life course stages the PBMA group would like to invest in. Priority areas were defined by the Welsh Government’s ‘Our Healthy Future’ report [19].

### Results of the marginal analysis task

We received 9 completed Marginal Analysis tasks from the panel and HIAG members out of a possible 30 responses. Though disappointing, the 9 respondents were representative of the wider panel with regards to their role and expertise spanning Public Health Wales, Welsh Government, Health Boards, local government and primary care.

As shown in Table 6, respondents allocated the largest proportion of the hypothetical £5 million to their first choice priority area and the smallest proportion to their third choice priority area. Only one respondent gave an equal division of the £5 million to each of the 3 rankings. Obesity was given the largest proportion of the £5 million, followed by: Mental Health and Wellbeing, Tobacco Control, Nutrition, Substance misuse, physical activity, Injuries and finally Oral Health. The rationale for respondents’ choices were mainly based upon the large adverse costs and impacts on the population of poor population health in these priority areas, with the potential for large benefit if these areas were given funding and priority. One respondent stated their decisions were based upon key local priorities. Another respondent stated their decisions were based on their view that two priority areas were generally underfunded, though could have wide ranging benefits.

**Table 6:** **Results of the marginal analysis ranking exercise for the 11 priority areas from the 9 respondents**

Priority area	Total number of times the area was assigned 1st ranking	Total number of times the area was assigned 2nd ranking	Total number of times the area was assigned 3rd ranking
Tobacco control	1	2	2
Physical activity	1	0	0
Nutrition	0	0	2
Oral health	0	0	1
Obesity	3	3	1
Substance misuse	0	1	1
Sexual health	0	0	0
Injuries	1	0	0
Mental health and wellbeing	3	2	1
Public health education	0	0	0
Work and health	0	1	1

**Figure 3:**
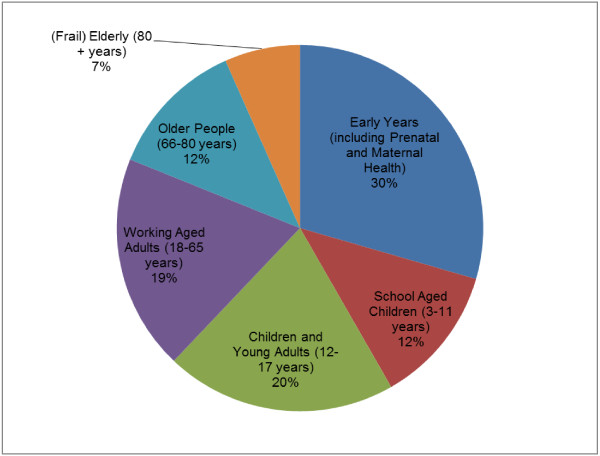
**Results of the marginal analysis exercise - proportion of the total allocation of the hypothetical £5 million for the 6 life course stages from the 9 respondents.**

As shown in Table 7 and Figure 3; the majority of respondents allocated the largest proportion of the hypothetical £5 million to their first choice life course stage and the smallest proportion to their third choice life course stage. However, one respondent allocated the largest proportion to their third ranking as they felt this life area was often neglected and underfunded. The rationale for respondents’ choices was mainly based upon the view that focusing on early intervention could provide the greatest potential to gain in the long-term. The early years life course stage received the highest number of first rankings. Respondents also stated that keeping older people healthy for as long as possible could have huge potential public health gains and savings to the NHS and social care sectors.

**Table 7:** **Results of the marginal analysis ranking exercise for the 6 life course stages from the 9 respondents**

Life course stage	Total number of times the stage was assigned 1st ranking	Total number of times the stage was assigned 2nd ranking	Total number of times the stage was assigned 3rd ranking
Early years (including prenatal and maternal health)	4	2	2
School aged children (3–11 years)	1	2	0
Children and young adults (12–17 years)	2	2	2
Working aged adults (18–65 years)	2	1	2
Older people (66–80 years)	1	0	2
(Frail) elderly (80 + years)	0	1	1

### Which NICE recommendations are not being implemented in Wales

As part of the next steps of the PBMA exercise and recommendations, it was stated that the evidence gathering exercise identified areas where Wales was not implementing existing NICE public health recommendations. Unlike NICE clinical guidelines, NICE public health guidance is not commissioned by the Welsh Government. Nonetheless, the recommendations of NICE are evidence based and would have similar value in Wales as in England. There is limited information on the extent of implementation of NICE public health guidance in Wales. There is potential for the introduction of systematic implementation and monitoring of NICE recommendations in Wales. There are a number of existing programmes where greater impact could be achieved through more systematic targeting and implementation, more robust monitoring and greater reach. There are a number of interventions with evidence of effectiveness not currently being implemented in Wales [see Additional file 3].

## Discussion

This PBMA exercise has generated practical policy lessons for Welsh Government, Public Health Wales and their partner agencies. Though PBMA has been applied in a range of clinical settings in the UK and internationally e.g., maternity services, it has not to our knowledge been used at a national level to review a whole programme of public health spending. The results from this PBMA exercise were used to inform the Public Health Wales report ‘Transforming Health Improvement in Wales’ which makes recommendations for reinvestment and disinvestment decisions and detailed actions for the future for health improvement in Wales [20]. The budget presented for this PBMA exercise was historical within the behest of the Minister for Health and Social Care for Wales. It was split in terms of 70% allocated via Public Health Wales and 30% directly allocated by Welsh Government. What became clear through the PBMA process was the importance of a programme having a logical and comprehensive boundary. The £15.1 million did not represent the total spending on health improvement across Wales and it is likely that many examples might be found of matched funds, through arrangements between Welsh Government, local Government, the voluntary sector and other agencies. It became difficult for the members of the PBMA group to comprehend the task of reallocating the £15.1 million without full knowledge of what resources, and what interventions, were being devoted to tackling health improvement issues outside the programme budget under review. The same argument could be made for previous published PBMA exercises. For example, Twaddle and Walker reviewed gynaecological services in Glasgow [14]. In hindsight, they may have benefited from consideration of a wider context of spending e.g. across primary care, other hospitals and other related agencies.

### QALYs in public health

Despite a growing view that QALYs may be an insufficient outcome measure to fully capture the benefits of public health interventions [5–7, 21], 85% of 200 cost per QALY estimates relating to NICE public Health Guidance produced a cost-per QALY of under the NICE threshold of £20,000 per QALY [3]. We found that in reviewing evidence of cost-effectiveness it was necessary to try to find common units of benefit with which to compare across a whole range of health improvement interventions. QALYs, Disability Adjusted Life Years (DALYs), and life years gained were most useful. What proved to be more difficult was the ability to use information on cost-effectiveness studies which used natural units of effect directly relevant to the public health intervention concerned (e.g., point change on a child behaviour index, minutes of exercise per week, number of smokers quitting). This PBMA exercise reinforced the argument for common units of benefit for the purpose of comparing across a whole programme of interventions, even in a public health setting.

We found few return on investment studies, or cost-benefit studies of public health improvement interventions. Placing monetary values on health outcomes, whether clinical or public health remains difficult, though cost-benefit analysis and cost-consequence analysis are recommended by NICE [22].

### Public health – invest to save

There is a growing interest amongst health care commissioners and local government for the concept of “invest to save” to be applied to public health interventions. This was also demonstrated by the PBMA panel in the marginal analysis task. The early years life course stage received the highest number of first rankings. The early years of the life course stages also received a higher proportion of the £5 million that later stages of life with reasons highlighted that focusing on early intervention could provide the greatest potential to gain in the long-term. It is worth noting there is no similar pressure for clinical services to be assessed in this way. From an economic perspective clinical and public health interventions can both be seen as having a common objective – to produce health benefits and the key issue should therefore be to identify which types of intervention produce the most health benefits and wider social benefits per £ on the margin. It is thus arguably disingenuous to demand that public health interventions demonstrate an “invest to save” benefit when we do not expect this of new drugs and surgical interventions in the NHS [23].

### Limitations of the PBMA exercise

This exercise was the result of a direct request from the Minister for Health and Social Care in Wales to review the specific health improvement budget of £15.1 million. This gave the exercise momentum; however, outside of these contexts where there is not high level political support, the generalisability of this PBMA exercise may be limited. It was recognised that across Welsh Government there were other diverse budgets that could be linked with health improvement activities e.g. through matched funding with local government and the voluntary sector. This meant that we were, at best, undertaking a “partial analysis”, and needed to keep in mind wider patterns of spending, as far as these could be identified in the time allowed. Common themes and concerns highlighted by the authors that emerged from the three PBMA sessions are summarised in Table 8.

**Table 8:** **Key themes and concerns emerging from the PBMA process and sessions as noted by the authors**

Number	Key themes and concerns
1	There is no readily available source of information on wider spending in Welsh Government and Public Health Wales on health improvement to provide the big picture context to the exercise.
2	It is very difficult to find evidence of effectiveness and cost-effectiveness relating specifically to different time horizons or national versus local provision.
3	The panel may need information about the proportion of the population who may take up a service when thinking about budget share i.e. population affected.
4	What is the (purpose/function) role of the “budget” i.e., the pot of money under consideration? What makes it different from other budgets /pots of money?
5	How we might best assess the effect of combined interventions and integrated approaches?
6	How we might best assess the effect of combined interventions and integrated approaches?
7	Government priorities can sometimes be based upon serial decision making rather than parallel decision making.

Another limitation of the PBMA exercise was the marginal analysis task. Due to time constraints the panel were unable to complete the task as part of the face to face group discussion sessions. Rather than omit this step, a pragmatic, high level e-mail based task was devised. This task was used to indicate the direction of travel and provide further recommendations for next steps; it is also worth noting a strength of this task was that it made the panel consider opportunity cost. However, only 9 PBMA panel members completed the marginal analysis task. Though these members were representative of the wider group with regards to role and expertise, the lack of response limits the potential wider applications of the recommendations given in this task. As the panel included front line clinical staff, we had 3 sessions to make participation in this PBMA exercise as manageable as possible given the work commitments of the members. As many PBMA exercises are shown to be unsuccessful [10] it was important that the panel completed all stages of the process, including a marginal analysis task though the limitations of the pragmatic task chosen here are noted above. The smaller response rate may be attributed to the fact that this task was conducted via e-mail rather than in person.

### Strengths of the PBMA exercise

This Health Improvement Review and the PBMA exercise offered the first transparent detailed breakdown of spending on 25 health improvement initiatives, within a ministerial budget. This provided a starting point for Welsh Government and Public Health Wales to expand the scope, if required, and gain a greater understanding of what is spent on health improvement in Wales. The review of initiatives allowed the panel to see what programmes are currently operating in Wales and suggest further improvements for initiatives (e.g., targeting), alternate delivery systems or new initiatives based upon NICE guidance. The PBMA exercise also generated a list of interventions recommended by NICE that are not currently being delivered in Wales as part of potential next steps and further research. A next step would be to generate evidence booklets for these, estimating what could be achieved with e.g., £1 million invested in any one of these new interventions. There would be a need to see how they would dove-tail with existing interventions and goals. Using the definitions of success categorised by Tsouparas and Frew [10] in their review, we argue our PBMA exercise would be considered successful in terms of the participants gaining a better understanding of the area under interest. It would also be considered successful as the recommendations were taken further in a report to the Minister, with changes to resource allocation made and the promotion of an evidence based culture in order to aid future resource allocation decisions.

## Conclusions

The PBMA exercise provided a useful platform to discuss and prioritise public health initiatives in Wales, taking account of the budget, their evidence base (including clinical effectiveness, cost-effectiveness and equity considerations), stakeholder views on and alternative options for delivery. The electronic voting on candidates for investment and disinvestment showed a clear recommendation for total disinvestment in 4 initiatives and a recommendation for partial disinvestment in 6 further initiatives due to lack of evidence for their effectiveness and cost-effectiveness at the time. The marginal analysis exercise indicated the direction of travel, the PBMA panel and HIAG group members advocated shifting funding to prioritise areas associated with large adverse health and social care costs. Priority was given to interventions that impact on sections of the population with the poorest health e.g., obesity and tobacco control. The panel also advocated focusing on early intervention, as this has the potential to result in large gain in the long-term. The evidence sub-groups were able to suggest which interventions Wales could be prioritising based upon NICE guidance. Wales spends a very small proportion of its NHS budget on health improvement. This exercise helped demonstrate the activity currently undertaken in the budget and its impact on the population, which was currently unknown. This was a necessary process to promote an evidence based culture to help resource allocation decisions, which has been promoted further since the completion of the exercise. Within the current climate of “prudent healthcare”, we have demonstrated that, at a National level, the PBMA process can reach decisions about potential candidates for disinvestment and potential investment in priority areas and life course stages. The next steps are to estimate the financial and health gain returns from reallocating resources released in this process.

## Electronic supplementary material

Additional file 1: **Methodology Booklet describing the process of evidence gathering, summarising and grading for each evidence stream.** (DOC 70 KB)

Additional file 2: **Brief descriptions of the 25 initiatives included in the health improvement review and PBMA exercise.** (DOC 48 KB)

Additional file 3: **Interventions with evidence of effectiveness recommended by NICE, that are not currently implemented in Wales.** (DOC 35 KB)
