# Supplementary material for: A national Programme Budgeting and Marginal Analysis (PBMA) of health improvement spending across Wales: disinvestment and reinvestment across the life course
Source: BMC Public Health. 2014 Aug 12;14:837. doi: 10.1186/1471-2458-14-837 (PMC4246570; doi:10.1186/1471-2458-14-837)
Supplement: Supplementary file 1 — Additional file 1: Methodology Booklet describing the process of evidence gathering, summarising and grading for each evidence stream. (DOC 70 KB) [file 12889_2014_7286_MOESM1_ESM.doc]

### Additional file 1 – Methodology Booklet describing the process of evidence gathering, summarising and grading for each evidence stream.

| **Initiative Description** | This is a narrative about the initiative (which was verified by the service provider) |
| --- | --- |
| **Priority area** | Each initiative was assigned to one of the priority areas from “Our Healthy Future”:  Tobacco Control /Physical Activity/ Nutrition & Oral Health /Obesity /Substance Misuse (alcohol & Drugs)/ Sexual Health / Injuries  Mental Health & Wellbeing / Work & Health / Public Health Education |
| **Life course stage** | Interventions were assigned to the following life-course stages:  Prenatal, Maternal Health and Early Years; School Child (3-11 years); Children & Young Adults; Working Age Adults (18-65 years); Older People (60-80 years); (Frail) elderly (80 +years) |
| **Intervention** | A descriptive narrative about the health improvement intervention delivered by this initiative ( an initiative categorised as having an intervention which seeks to influence a behavioural or health outcome, through direct or indirect action) |
| **‘total’ cost 2012/13** | Total cost for 2012/2013 as supplied by Finance Departments of WG and PHW. Source of funding identified as WG or PHW |
| **Evidence of effectiveness** | An ‘Initiative Assessment Log’ was completed for each initiative. Information from any evaluation or other reports about the initiative in Wales was considered for relevance and pertinent information summarised into the log along with the Summary Evidence Grade and other information.  A final ‘Initiative Grade’ was then applied by one reviewer using set criteria (Table 1). This takes into account both the research evidence of potential effectiveness and evidence of actual effectiveness in Wales, where available. Initiative grades were checked for consistency by comparison and discussion amongst the review team.  **Table 1:**   | **Initiative Grade^1^:** | **G I** | **G II** | **A I** | **A II** | **A III** | **R I** | **R II** | **R III** | **R IV** | | --- | --- | --- | --- | --- | --- | --- | --- | --- | --- | | **Priority area?** | **Yes** | **Yes** | **Yes** | **Yes** | **Yes** | **Yes** | **Yes** | **Yes** | **N** | | **Summary Evidence Grade^2^** | **++** | **+** | **++** | **+** | **+/-**  **or**  **0** | **+/-**  or  **0** | **-or- -** | **-or- -** | **N.A** | | **Evaluation^3^?** | **Yes** | **Yes** | **No** | **No** | **Yes** | **No** | **Yes** | **No** | **N.A.** |   1. G=Green, A=Amber, R=Red N.A. = Not applicable  2. Overall grade for evidence-base for initiative  3. If there is no local (Welsh) evaluation or if the evaluation does not provide information about achievement of appropriate outcomes (effectiveness), then record ‘No’. |
| **Research evidence of Cost effectiveness** | Relevant articles identified from an evidence search (2012-2002) of NICE, Pub-Med and the Centre for Reviews and Dissemination (CRD) Database using key terms from each of the 43 Priority area programmes were sourced and then appraised as follows:  Evidence was defined as;   1. **Directly relevant** i.e. an economic evaluation of a specific intervention delivered through the programme/initiative stated in the list of included programmes 2. **Indirectly relevant** (where directly relevant evidence is unavailable) i.e. evaluation of related intervention similar to the one delivered through the programme/initiative or as part of the intended aims of the programme/initiative stated in the list of included programmes by either method of delivery (school-based smoking cessation) or target population (pregnant women).   The Drummond et al. (2005) checklist for a sound economic evaluation was used to appraise evidence found in the electronic searches. A subjective judgement of the overall balance of economic evidence was made by the economic evidence sub-group and the following traffic light system of grading was used.   | **Green I** | **Green II** | **Amber I** | **Amber II** | **Red I** | **Red II** | | --- | --- | --- | --- | --- | --- | | **Large frequency of good quality economic evaluations showing cost-effectiveness/ cost-savings/ cost-benefits as appraised by Drummond et al’s 2005 Checklist for a sound economic evaluation** | **Small frequency of good quality economic evaluations showing cost-effectiveness/ cost-savings/ cost-benefits as appraised by Drummond et al’s 2005 Checklist for a sound economic evaluation** | **Fair quality economic evaluations showing cost-effectiveness/ cost-savings/ cost-benefits as appraised by Drummond et al’s 2005 Checklist for a sound economic evaluation** | **Fair quality evidence however, showing mixed evidence of cost-effectiveness/ cost-savings/ cost-benefits as appraised by Drummond et al’s 2005 Checklist for a sound economic evaluation** | **Poor quality evidence showing intervention was not cost-effectiveness/ cost-savings/ cost-benefits as appraised by Drummond et al’s Checklist for a sound economic evaluation** | **No evidence available** | |
| **Reach** | Information obtained from direct contact with programme providers or commissioners. Where possible the % of population reached was calculated. |
| **Inequalities** | Categories The following categories have been developed to grade the degree of equality/equity focus of each programme:  White  The initiative is not expected to impact on inequalities  The initiative is specifically designed to reduce health inequalities or this is one of its stated or implicit aims and there is evidence of this.  Green  Amber  The initiative has reducing health inequalities/inequities stated as a specific aim, but there is no evidence or some evidence that this is likely to be the case.  Red  The initiative has no stated impact on or discernible features likely to reduce health inequalities/inequities.  Some of these programmes have a degree of complexity which require a few sentences of explanation in addition to the colour coding. These include some where there has been a change of focus since inception and others where programme employees act as intermediaries and local areas are largely autonomous in the way programmes are delivered.  It should be noted that these categories apply to the *intention* of the programme rather the supporting evidence, effectiveness or cost effectiveness, which have been reviewed separately. |
| **Mechanisms of delivery** | Delivery method and setting |
| **Wider views & suggestions for alternative delivery from stakeholders** | *Local Public Health Teams:* Semi structured meetings were held with each of the seven LPHT’s. Sessions were based around eight broad questions. The sessions did not ask the teams to make specific comment on each of the initiatives under review.   - Where >3 LPHT’s positively commented on a specific initiative it was classified **GREEN** - Where <3 LPHT’s negatively commented on a specific initiative it was classified **RED** - Anything else mentioned was classified as **AMBER**   *Public Engagement:* Beaufort Research were commissioned to undertake a public survey and to conduct six focus groups and six in-depth family interviews.   - Where >70% of a group positively commented on a specific initiative it was classified GREEN - Where <30% of a group negatively commented on a specific initiative it was classified RED - Anything else mentioned was classified as AMBER   *Online form*: An online feedback form was hosted on the bilingual review web pages on the Public Health Wales website. It was based around four broad questions.   - Where >70% of responders positively commented on a specific initiative it was classified GREEN - Where <30% of responders negatively commented on a specific initiative it was classified RED - Anything else mentioned was classified as AMBER |
| **Policy Link(s)** | Relevant policy context |
