# Supplementary material for: A national Programme Budgeting and Marginal Analysis (PBMA) of health improvement spending across Wales: disinvestment and reinvestment across the life course
Source: BMC Public Health. 2014 Aug 12;14:837. doi: 10.1186/1471-2458-14-837 (PMC4246570; doi:10.1186/1471-2458-14-837)
Supplement: Supplementary file 2 — Additional file 2: Brief descriptions of the 25 initiatives included in the health improvement review and PBMA exercise. (DOC 48 KB) [file 12889_2014_7286_MOESM2_ESM.doc]

Additional File 2 Brief description of the 25 initiatives included in the health improvement review and PBMA exercise.

| **Initiative** | **Brief Description of Initiative** |
| --- | --- |
| **Cooking Bus** | The Cooking Bus is an articulated lorry with 4 fully fitted kitchens and teaching staff providing 90 minute lessons covering food preparation, cooking skills and food hygiene for 16 Primary School pupils at a time. The Bus visits 45-50 Primary schools per year. |
| **MEND** | This is an intensive parent and child obesity intervention, for children who are overweight, with the aim of reducing BMI through a group based programme. |
| **Mental Health First Aid** | This is a Third Sector delivered, 12 hour training course that improves mental health literacy and helps people to recognise the signs and symptoms of someone with mental health problems; to respond to various mental health crises, engage with, support and signpost people to appropriate help. |
| **Smokebugs** | This is a programme targeted at 8-11 year olds to raise awareness of the dangers of smoking and reduce the number of smokers along with encouraging and supporting those who wish to remain non-smokers. |
| **National Breastfeeding Programme – Breastfeeding Peer Support Programme (BPSP)** | This programme involves the recruitment and training of peer supporters for breast feeding mothers, provision of accessible local peer support and groups, which have established throughout Wales. |
| **National Breastfeeding Programme – Breast-feeding Welcome Scheme (BFWS)** | This scheme aims to promote the cultural acceptance of the importance of breastfeeding and thus seeks to contribute to the reduction of barriers to continued breastfeeding by enabling mothers to breastfeed when and where they need to. The scheme has been established by the Welsh Government to identify premises that understand and support the needs of breastfeeding mothers and their babies. |
| **Health Challenge Wales website**  **Cost** | Health Challenge Wales is a healthy lifestyle campaign launched in 2004. This website allows information to be presented by this campaign and other relevant programmes and campaigns. |
| **Smokers Helpline** | This helpline is provided through NHS Direct and provides callers with basic information and signposting on smoking cessation. The service is bilingual and runs from 09:00-19:00 daily. |
| **Smoking Resources** | This initiative produces leaflets for smoking related initiatives. |
| **Skin Cancer Awareness** | This is a skin cancer awareness website with downloadable leaflets and posters in Welsh and English to promote skin cancer prevention messages and raise awareness of symptoms. |
| **Designed to Smile** | This multi-component programme includes oral health education delivered by health visitors, supervised tooth brushing training and supply of tooth brushes for 3-5 year olds, fluoride varnish and oral health education for 6-11 year olds. |
| **Welsh Network of Healthy Schools Schemes** | This is an award based initiative which recruits schools who then commit to undertake a series of actions to promote health. Schools identify their own priorities and develop an action plan; achievement of this plan is assessed locally. The programme emphasises the creation of an environment within schools that is supportive of health. |
| **Stop Smoking Wales – Pre Surgery** | Smokers undergoing elective surgery are encouraged to stop smoking four weeks prior to surgery and to encourage longer term cessation as a secondary goal. |
| **Stop Smoking Wales – Pregnancy** | Pregnant women who wish to stop smoking are providing with support and access to services to quit smoking whilst pregnant and are encouraged to quit smoking longer term. |
| **Stop Smoking Wales – Vulnerable Groups** | Support is provided to workers in key settings e.g. prisons and mental health inpatient facilities, including training and development of a manual to enable them to deliver smoking cessation. |
| **Stop Smoking Wales – Brief Intervention Training** | Training is provided for health and other professionals to be able to give brief advice to individuals who wish to quit smoking and refer to further support. |
| **Fresh Start Wales** | This programme aims to raise awareness of the risks of second hand smoke, particularly in cars, behaviour change and generate public support for legislative change, if needed through a mass media campaign. |
| **Alcohol Brief Interventions in Primary Care Training** | Training is provided to increase the use of assessment of drinking habits using a recognised tool and where appropriate provide brief intervention for alcohol in health, social care and community settings. |
| **HIV Prevention** | This is a HIV awareness raising campaign in community settings aimed at the general population followed by targeted campaign tools for high risk groups including young people and men who have sex with men |
| **National Exercise Referral Scheme** | This is a programme of supervised exercise (32 sessions over 16 weeks), tailored to participants’ condition. The goal is to increase levels of physical activity. The programme seeks to reduce barriers to exercise by subsidising cost, reducing anxiety or unfamiliarity with leisure or exercise settings and provide instruction in the use of exercise equipment. Individuals are usually referred by their GP to this initiative. |
| **Stop Smoking Wales – Adults** | An intensive six week behavioural support with support to access pharmacotherapy for smokers delivered in community settings. The target group is usually older smokers who have already had several quit attempts. |
| **ASSIST** | A school based programme for 12-13 year olds that trains peers to work with individuals through a central specialist team. The training encourages pupils to engage in discussion with peers to promote smoke free lifestyles. |
| **National Breastfeeding Programme -**  **Baby Friendly initiative (BFI)** | This initiative provides training for health professionals to enable them to give breastfeeding mothers the help and support they need to breastfeed successful. |
| **No Smoking Day** | This is an annual event targeted at smokers to encourage a quit attempt. |
| **Teenage Pregnancy Pilot** | This is a pilot project to reduce unwanted teenage pregnancies by providing education and raising awareness of the benefits of Long Acting Contraception with particular emphasis on Looked After Children and Care leavers in selected areas of Wales. |
| **Steroids and Image Enhancing Drugs** | This is a training and awareness programme for those working with and those using steroids and image enhancing drugs. The programme includes, a website, a film providing safer injecting information, information available via the website and in hard copy and an accredited training course. |
| **Champions for Health** | This campaign is aimed at NHS employees to provide an opportunity to improve their health and act as ambassadors to their patients and the public on the importance of leading a healthy lifestyle and the benefits it brings. |
