# Supplementary material for: A national Programme Budgeting and Marginal Analysis (PBMA) of health improvement spending across Wales: disinvestment and reinvestment across the life course
Source: BMC Public Health. 2014 Aug 12;14:837. doi: 10.1186/1471-2458-14-837 (PMC4246570; doi:10.1186/1471-2458-14-837)
Supplement: Supplementary file 3 — Additional file 3: Interventions with evidence of effectiveness recommended by NICE, that are not currently implemented in Wales. (DOC 35 KB) [file 12889_2014_7286_MOESM3_ESM.doc]

Additional File 3 Interventions with evidence of effectiveness recommended by NICE, that are not currently implemented in Wales.

| **Health Topic** | **Intervention Recommended by NICE not Currently implemented in Wales** |
| --- | --- |
| **Smoking cessation** | - Electronic Health Record Interventions - The provision of smoking cessation in the workplace. - Nursing interventions (dedicated rather than routine interventions more effective) - Telephone counselling, there is potential to extend the existing smokers helpline and offer pro-active counselling as well as reactive - Mass media interventions for smoking cessation in adults - Targeted programmes at disadvantaged groups |
| **Smoking Prevention** | - Mass media interventions for smoking prevention with young people |
| **Physical Activity** | - NICE guidance recommends training health professionals (and other health and fitness advisors) to give advice to pregnant women about physical activity. - Whole-setting approach to physical activity in child-care settings - There is some evidence to suggest that brief interventions about physical activity in primary care can be effective, particularly when intensive, followed-up and targeted at the highest risk individuals. - NICE guidance about the importance of planning and development of infrastructure (roads, towns, schools, public places etc) to promote active travel, active play and provision of safe, welcoming, acceptable and appropriate facilities for physical activity - Whole setting approaches in Workplaces |
| **Nutrition** | - Intensive dietary counselling in primary care for high risk individuals - Workplace approaches |
| **Obesity** | - Worksite behaviour modification programmes, that include health screening with counselling/education can result in short-term weight loss although weight loss may be regained post intervention. - Sustained health-professional-led interventions in primary care or community settings, focusing on diet and physical activity or general health counselling can support maintenance of a healthy weight. - Interactive computer-based interventions can be an effective intervention for weight loss and weight maintenance but are less effective than in-person interventions. |
